# Role of cardiac mitofusins in cardiac conduction following simulated ischemia–reperfusion

**DOI:** 10.1038/s41598-022-25625-0

**Published:** 2022-12-06

**Authors:** Xiu-Yi Kwek, Andrew R. Hall, Wei-Wen Lim, Khairunnisa Katwadi, Poh Loong Soong, Elina Grishina, Kun-Han Lin, Gustavo Crespo-Avilan, En Ping Yap, Nur Izzah Ismail, Kroekkiat Chinda, Ying Ying Chung, Heming Wei, Winston Shim, David Montaigne, Andrew Tinker, Sang-Bing Ong, Derek J. Hausenloy

**Affiliations:** 1 National Heart Research Institute Singapore, National Heart Centre, Singapore, Singapore; 2 The Hatter Cardiovascular Institute, Institute of Cardiovascular Science, University College London, London, UK; 3 Cardiovascular and Metabolic Disorders Program, Duke-National University of Singapore Medical School, Singapore, Singapore; 4 Yong Loo Lin School of Medicine, National University Singapore, Singapore, Singapore; 5 Cardiovascular Translational Program, Cardiovascular Research Institute (CVRI), National University of Singapore, Singapore, Singapore; 6 Department of Medicine, National University Hospital of Singapore (NUHS), Singapore, Singapore; 7 Ternion Biosciences, Singapore, Singapore; 8 Department of Biochemistry, Medical Faculty, Justus Liebig-University, Giessen, Germany; 9 Centre for Cardiovascular Genomics and Medicine (CCGM), Lui Che Woo Institute of Innovative Medicine, Chinese University of Hong Kong (CUHK), Hong Kong, SAR China; 10 Department of Medicine and Therapeutics, Faculty of Medicine, Chinese University of Hong Kong (CUHK), Hong Kong, SAR China; 11 Hong Kong Hub of Paediatric Excellence (HK HOPE), Hong Kong Children’s Hospital (HKCH), Kowloon Bay, Hong Kong, SAR China; 12 Department of Physiology, Faculty of Medical Science, Naresuan University, Phitsanulok, Thailand; 13 Integrative Cardiovascular Research Unit, Faculty of Medical Science, Naresuan University, Phitsanulok, Thailand; 14 Centre for Vision Research, Duke-National University of Singapore Medical School, Singapore, Singapore; 15 Research Laboratory, KK Women’s & Children’s Hospital, Singapore, Singapore; 16 Health and Social Sciences Cluster, Singapore Institute of Technology, Singapore, Singapore; 17 Inserm, CHU Lille, Institut Pasteur Lille, U1011-European Genomic Institute for Diabetes (EGID), University of Lille, 59000 Lille, France; 18 Centre for Clinical Pharmacology, William Harvey Research Institute, Barts and The London School of Medicine and Dentistry, Queen Mary University of London, Charterhouse Square, London, UK; 19 Joint Laboratory of Bioresources and Molecular Research of Common Diseases, Kunming Institute of Zoology-The Chinese University of Hong Kong (KIZ-CUHK), Chinese Academy of Sciences, Kunming, Yunnan China; 20 Shenzhen Research Institute (SZRI), Chinese University of Hong Kong (CUHK), Shenzhen, China

**Keywords:** Mechanisms of disease, Cardiovascular biology

## Abstract

Mitochondrial dysfunction induced by acute cardiac ischemia–reperfusion (IR), may increase susceptibility to arrhythmias by perturbing energetics, oxidative stress production and calcium homeostasis. Although changes in mitochondrial morphology are known to impact on mitochondrial function, their role in cardiac arrhythmogenesis is not known. To assess action potential duration (APD) in cardiomyocytes from the Mitofusins-1/2 (Mfn1/Mfn2)-double-knockout (Mfn-DKO) compared to wild-type (WT) mice, optical-electrophysiology was conducted. To measure conduction velocity (CV) in atrial and ventricular tissue from the Mfn-DKO and WT mice, at both baseline and following simulated acute IR, multi-electrode array (MEA) was employed. Intracellular localization of connexin-43 (Cx43) at baseline was evaluated by immunohistochemistry, while Cx-43 phosphorylation was assessed by Western-blotting. Mfn-DKO cardiomyocytes demonstrated an increased APD. At baseline, CV was significantly lower in the left ventricle of the Mfn-DKO mice. CV decreased with simulated-ischemia and returned to baseline levels during simulated-reperfusion in WT but not in atria of Mfn-DKO mice. Mfn-DKO hearts displayed increased Cx43 lateralization, although phosphorylation of Cx43 at Ser-368 did not differ. In summary, Mfn-DKO mice have increased APD and reduced CV at baseline and impaired alterations in CV following cardiac IR. These findings were associated with increased Cx43 lateralization, suggesting that the mitofusins may impact on post-MI cardiac-arrhythmogenesis.

## Introduction

Cardiac arrhythmias are a major cause of morbidity and mortality in acute myocardial infarction (MI). Acute cardiac ischemia and reperfusion (IR) following acute MI results in gap junction uncoupling and ion channel remodeling, culminating in perturbed cardiac conduction and increased risk of fatal and non-fatal cardiac arrhythmias^1^. Disturbances in action potential duration (APD) and cardiac conduction velocity (CV) are known to impact on cardiac arrhythmogenesis^2,3^. The speed of propagation of the depolarization wave across the myocardium relies primarily on gap junction conductivity^4^. Gap junctions comprise connexin (Cx) proteins which function to transmit electrical excitability^5^, with perturbations to the connexin proteins and gap junction function slowing down cardiac conduction, causing irregular propagation of the action potential by allowing wave-fronts to re-enter and re-excite regions asynchronously, predisposing to cardiac arrhythmias^6^.

During acute cardiac ischemia, the occurrence of mitochondrial dysfunction impairs respiration and ATP production, impacting on the function of cardiac ATPase (e.g. SERCA and Na–K ATPase), resulting in ionic imbalances, thereby predisposing to arrhythmias^7^. This is further exacerbated upon reperfusion when the production of reactive oxygen species (ROS) alters the activity of the L-type Ca^2+^ channel, Na–K ATPase and SERCA^8–10^. Connexin-43 (Cx43) is a major phosphoprotein of cardiac gap junctions which normally translocates between the sarcolemma and the cytosol^11^. Cx43 is regulated by de/phosphorylation by different kinases such as Protein Phosphatase1 (PP1)/PKA, protein kinase B (PKB or AKT), protein kinase C (PKC), calcium/calmodulin kinase II (CamKII), mitogen-activated protein kinase (MAPK), and the pp60src kinase (src)^12^. Ischemia reduces Cx43 phosphorylation thereby prompting Cx43 to translocate to the cytosol and reduces cardiac CV^13^. During reperfusion, Cx43 translocates back to the gap junctions in a PP1-dependent manner to recover CV^13^. However, elevated ROS at the onset of reperfusion may affect PP1 activity causing Cx43 to remain in its dephosphorylated form in the cytosol, leading to persistent impaired cardiac CV^14,15^.

Mitochondrial function has been established to play an important role in governing cardiovascular health. In this regard, changes in mitochondrial dynamics via the processes of fusion and fission are needed to ensure optimal mitochondrial function^16,17^. The fusion of two individual mitochondrion via the pro-fusion proteins such as the Mitofusins 1 and 2 (Mfn1, Mfn2) and optic atrophy protein-1 (OPA1) allows the replenishment of damaged mitochondrial DNA^18^. The fragmentation of mitochondria via the pro-fission protein Dynamin-related protein 1 (Drp1) is required for selective removal of damaged mitochondria by mitophagy^19^. The reduced expression of either Mfn1 or Mfn2 contributes to cardiac pathologies. Decreased Mfn1 as well as Mfn2 is known to contribute to mitochondrial fragmentation resulting in impaired metabolism and increased ROS in the settings of diabetes^20–23^. The reduction of Mfn2 expression is also observed in the hypertrophic heart^24^. The genetic ablation of both Mfn1 and Mfn2 (Mfn-DKO) has been previously demonstrated to cause mitochondrial fragmentation, impairment of mitochondrial function, and cardiac dysfunction^25^, with sudden death at 8 weeks^25^. Drp-1 mediated mitochondrial fission has been associated with cardiac ischemia–reperfusion injury which can be prevented directly by manipulating mitochondrial morphology^26,27^ or indirectly via manipulating the upstream pathways such as calcium regulation^28^.

Although changes in mitochondrial morphology are known to impact on mitochondrial function, their role in cardiac arrhythmogenesis is beginning to receive attention. The role of Mfn1 as a substrate for cardiac arrhythmogenesis has been recently exemplified using the endomyocardial biopsy of intraventricular septum from non‑responding patients (heart failure patients who received optimal conventional multidisciplinary therapy yet do not show favorable improvement) with idiopathic dilated cardiomyopathy (left ventricular (LV) ejection fraction (LVEF) of < 10% improvement on follow-up ultrasound cardiography performed at 7 and 15 months after biopsy) whereby Mfn1 was found to be significantly reduced by concomitant activation of adrenergic signaling and elevated miR-140-5p, albeit the level of Mfn2 remains unchanged. This observation was also corroborated by both in vitro depletion of the Mfn1 gene as well as isoproterenol administration whilst cardiac-specific deletion of Mfn1 promoted cardiac dilatation with reduced systolic function in mice subjected to LV pressure overload^29^.

With regards to Mfn2, genetic ablation of Mfn2 in the heart has been found to promote a delay in Ca^2+^-induced MPTP opening, in line with increased tolerance to local generation of ROS as well as a reduction in Mfn2-facilitated Ca^2+^ transfer from the ER to the mitochondria^30,31^. Although the intact Mfn-2 null heart exhibit mostly normal physiological function, isoproterenol administration in the Mfn-2 null myocytes induces a mild systolic dysfunction, which is mirrored by a minor decrease in contractility when paced ex vivo—characteristics of which can be attributed to a reduction in baseline mitochondrial membrane potential^21,22,32^. In the ventricular myocardium of a mouse model of aortic stenosis, aberrant mitochondrial fission indicative of excessive mitophagy and reduced Mfn2 contribute to abnormal activation of MMP-9, culminating in Cx-43 degradation, increased fibrosis and subsequent ventricular dysfunction^33,34^.

Although Mfn-DKO causes embryonic lethality, genetic inactivation of both Mfn1 and Mfn2 during the midgestational period prompted an increase in mitochondrial numbers at birth, which then further expand abnormally by postnatal day 7, with a high frequency of spherical mitochondria coupled with disrupted membrane and cristae organization. Myofibrils appear to be displaced by the mitochondria, with an increase in distance between parallel myofilaments. Wall motion in the DKO hearts was impaired, blood flow in the LV disrupted, heart rate was lower, and fractional shortening markedly decreased. More importantly, the QRS complex in the DKO hearts was significantly altered—a hallmark feature of dilated cardiomyopathy which causes death by postnatal day 16^35^.

In the adult heart, tamoxifen-induced conditional genetic ablation of both mitofusins causes a progressive dilation in the first 6 weeks with ensuing heart failure by the 7th and 8th week, albeit the exact association with cardiac arrhythmias remains to be investigated. The deleterious effect of the adult Mfn-DKO hearts has been attributed to an impaired mitochondrial fusion machinery and compromised mitochondrial respiration, although changes in other mitochondria-shaping proteins such as OPA1 and Drp1 were insignificant^25^.

In this research study, we assess cardiac APD and CV in Mfn-DKO mice at baseline and following simulated IR as an indicator of susceptibility to cardiac arrhythmias, and investigate the localization of Cx43 as an indicator of gap junction function.

## Results

### Cardiac-specific Mfn 1 and Mfn 2 deletion in the Mfn-DKO mice

**Figure 1:**
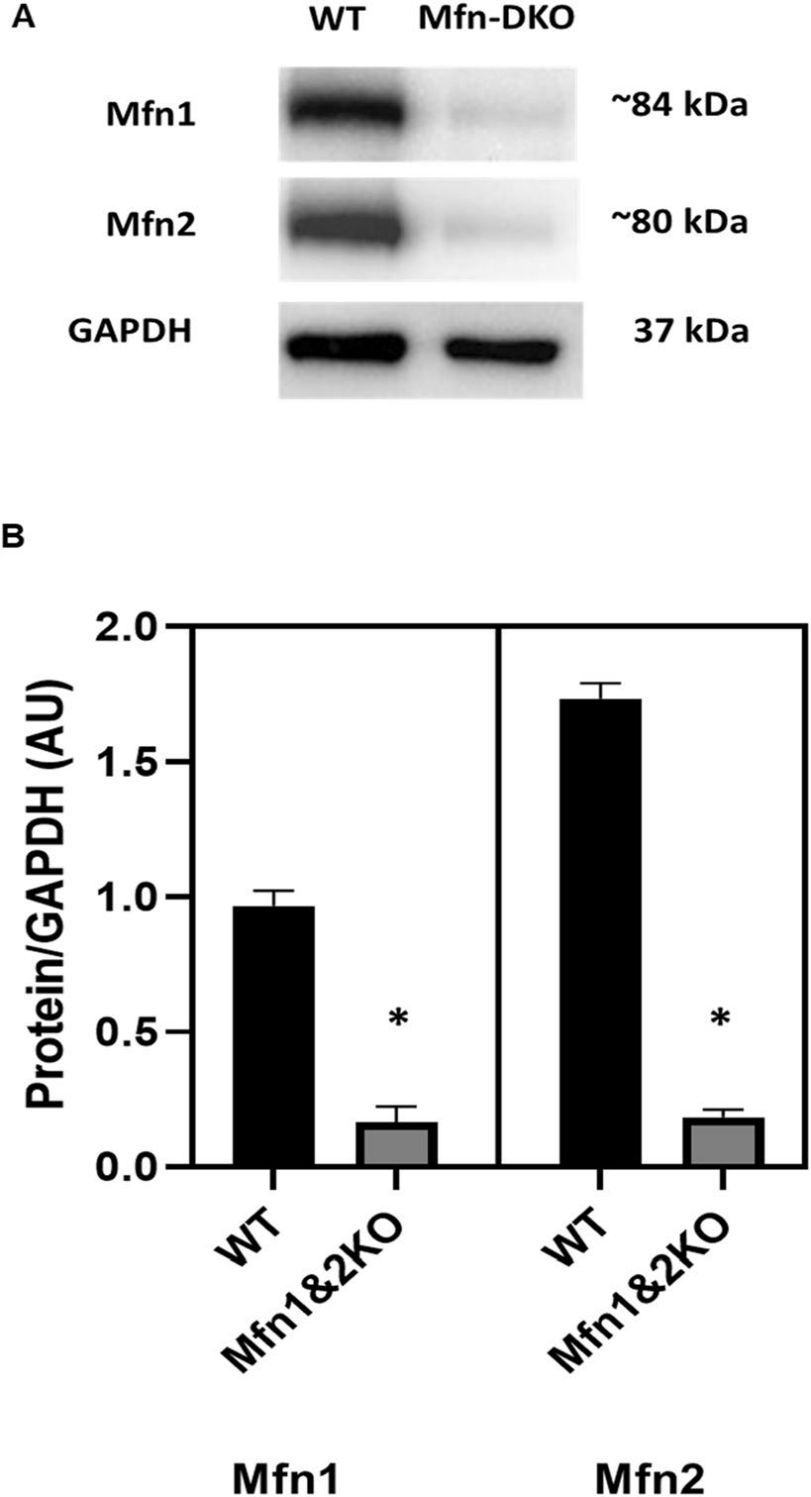
Mfn1 and Mfn2 expression in the Mfn-DKO hearts. (**A**) Representative Western blot image (cropped) of Mfn1 and Mfn2 expression in the WT control versus Mfn-DKO hearts. Original gel images are presented in Supplementary Fig. 1. (**B**) Densitometry analysis of Mfn1 and Mfn2 expression in the Western blot images; n = 3 hearts per group, *p < 0.05 when compared against WT.

To validate that both Mfn 1 and Mfn 2 are knocked-out in the hearts following tamoxifen injection, Western blot was performed on cardiac tissue of 10 weeks old mice. The expression of Mfn1 (~ 84 kDa) and Mfn2 (~ 80 kDa) significantly decreased in the hearts of Mfn-DKO mice compared to the WT mice (Fig. 1A,B).

### Increased action potential duration in the Mfn-DKO hearts

**Figure 2:**
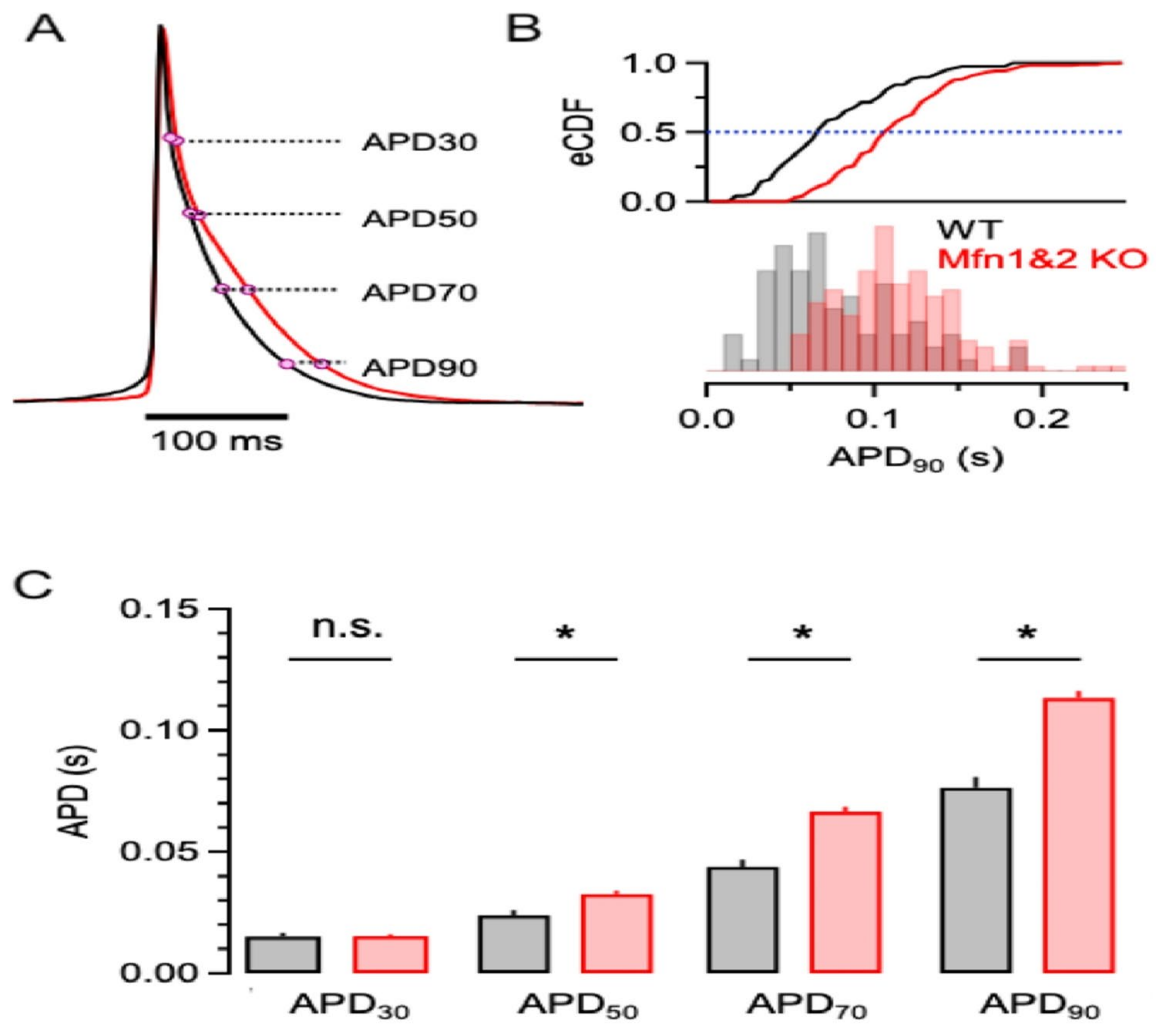
Functional characterization of isolated cardiomyocytes from mouse whole hearts using (OptioQUANT) platform. (**A**) Representative waveforms of action potential obtained from the wild-type (black trace) and Mfn1&2 KO (red traces) mouse cardiomyocytes. Dots represent AP points at 30%, 50%, 70% and 90% repolarization after depolarization. (**B**) Histograms (bottom) of wild type and mfn1&2 KO mouse cardiomyocytes show substantial variability and non-identical distribution of APD_90_. The empirical cumulative distribution plot (upper) shows prolonged action potential durations in the transgenic KO mouse cardiomyocytes. The blue dotted line illustrates median value. (**C**) Bar graph summarizing the APD at 30%, 50%, 70% and 90% of repolarization in WT (n = 77) and DKO (n = 152) cells. APD_30_: 0.015 ± 0.002 vs 0.015 ± 0.001; APD_50_: 0.024 ± 0.002 vs 0.033 ± 0.002; APD_70_: 0.044 ± 0.003 vs 0.066 ± 0.002 and APD_90_: 0.076 ± 0.004 vs 0.113 ± 0.003. All APD values in seconds. *p < 0.001 determined by Student's t-test. *eCDF: empirical cumulative distribution function; *APD* action potential duration.

The Mfn-DKO myocytes demonstrated an increase in mean average APD following a 50%, 70% and 90% decrement of AP amplitude (Fig. 2A,C). The empirical cumulative distribution plot (Fig. 2B—upper) shows prolonged APD in the transgenic KO mouse cardiomyocytes, mirroring the distribution of APD_90_ among the myocytes from WT and Mfn-DKO hearts (Fig. 2B—lower).

### Reduced conduction velocity in the Mfn-DKO hearts

**Figure 3:**
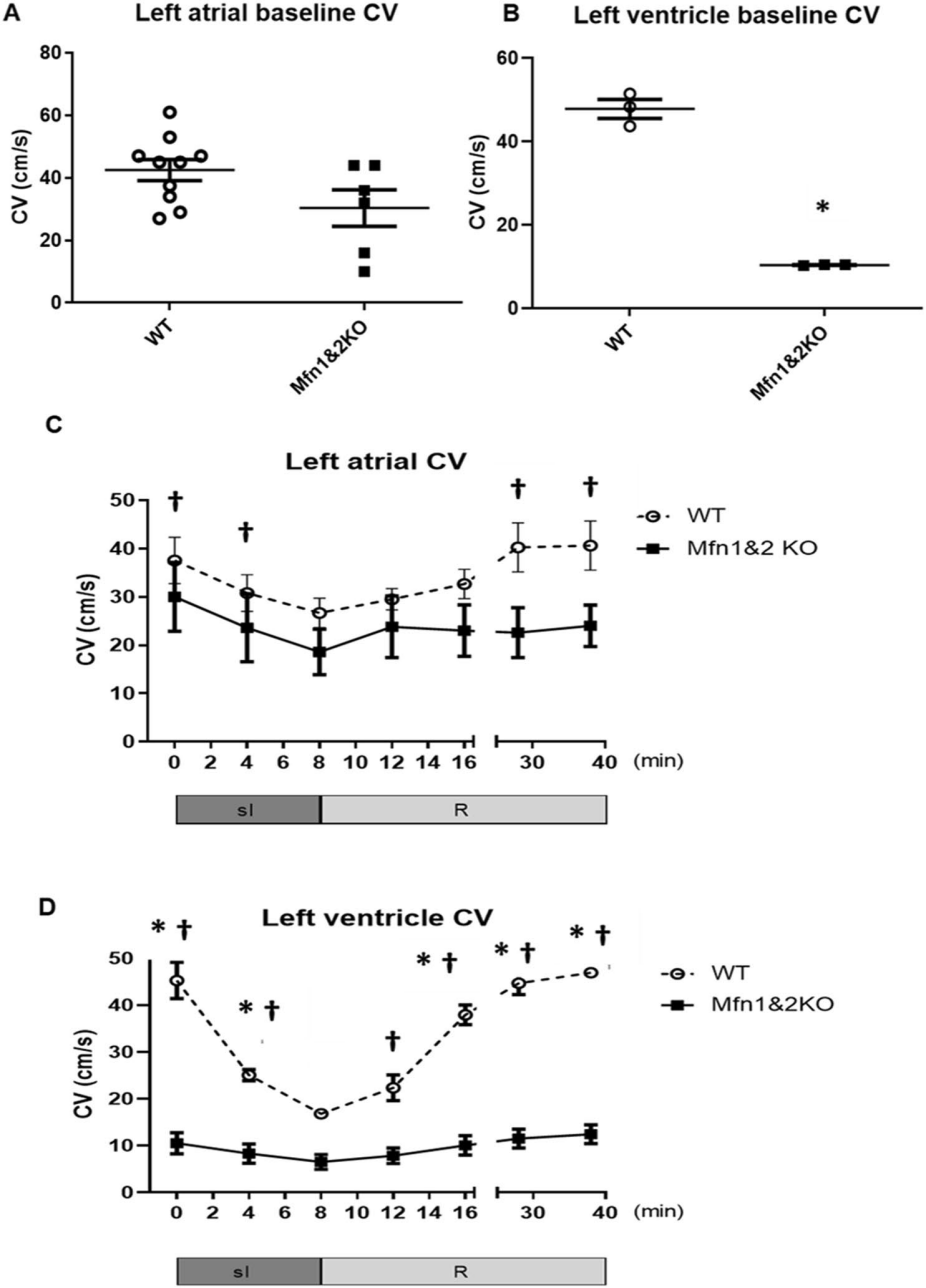
Conduction velocities (CV) of the atrial and ventricular tissue. Baseline CV of the (**A**) left atrial and (**B**) left ventricle of the wild-type (WT) versus Mfn-DKO hearts, *p < 0.05 when compared against WT. Changes in the CV during simulated ischemia (sI) and reperfusion (R) of the (**C**) left atrial tissue and (**D**) left ventricular tissue of the WT versus Mfn-DKO hearts, *p < 0.05 when compared against DKO; ^†^p < 0.05 when compared against the lowest CV at the end of ischemia. Data are presented as mean ± SEM.

Left atrial CV in Mfn-DKO hearts was reduced, albeit not statistically significantly, when compared to WT hearts (42.6 ± 3.4 cm/s in the WT hearts vs 30.3 ± 5.9 cm/s in the Mfn-DKO hearts, p-value = 0.071) (Fig. 3A). In left ventricular tissue, baseline CV in the Mfn-DKO hearts was significantly lower than that of WT hearts (47.9 ± 2.3 cm/s vs 10.4 ± 0.1 cm/s) (Fig. 3B). Upon subjecting the left atrial tissue to simulated ischemia on the MEA there was a significant decline in CV of the left atria of the WT hearts. Upon reperfusion, the CV increased to a level that is significantly higher than the lowest level of CV at the end of ischemia in the WT hearts but not in the left atria of Mfn-DKO hearts (Fig. 3C). In left ventricular tissue, simulated ischemia significantly reduced CV in the WT hearts but not the Mfn-DKO hearts. The CV recovered to pre-ischemia levels in both the WT and Mfn-DKO ventricular tissue during reperfusion, although the CV remained significantly lower in the left ventricle of Mfn2-DKO hearts compared to WT (Fig. 3D).

### Lateralization of Cx43 in the Mfn-DKO hearts

**Figure 4:**
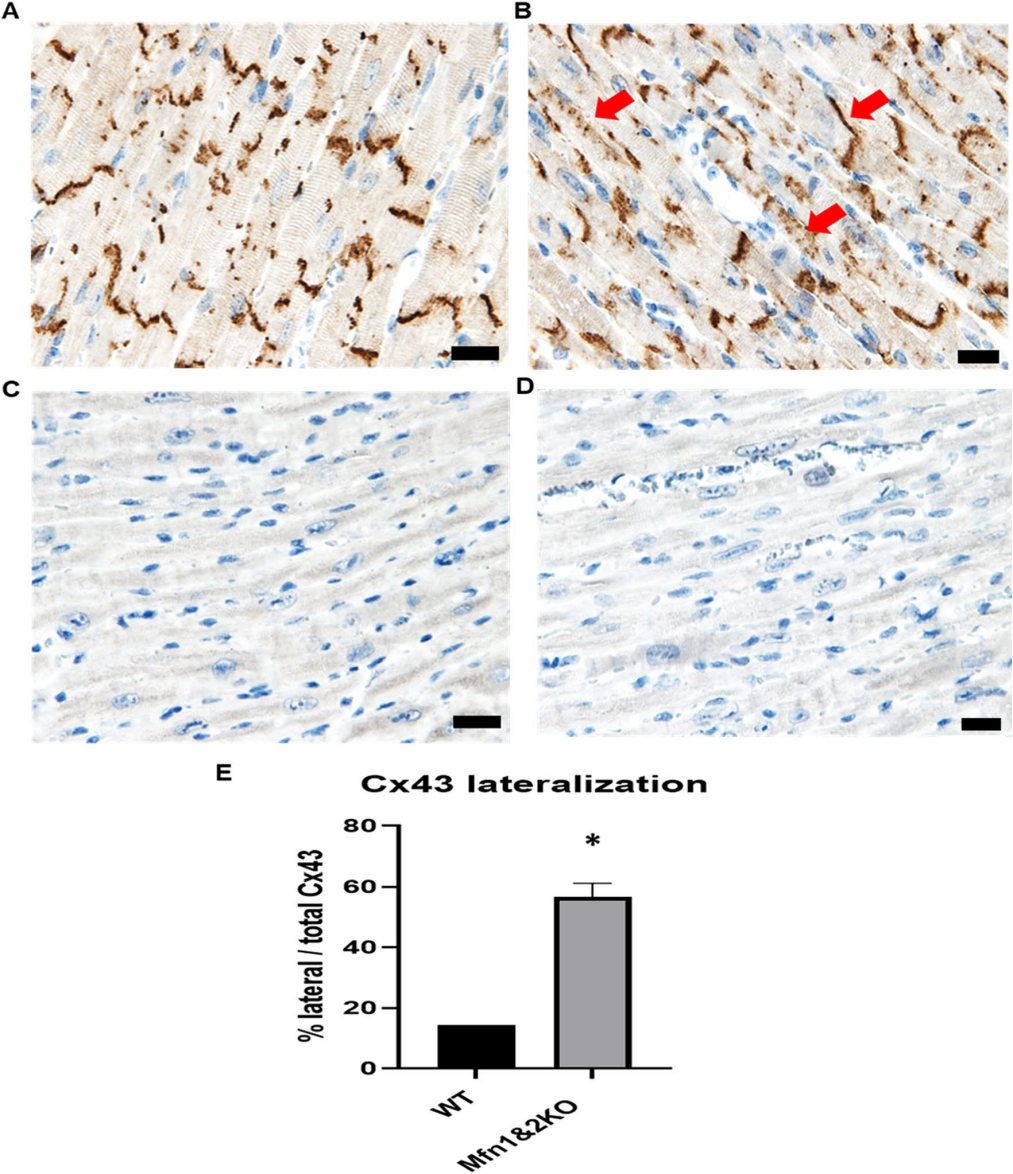
Lateralization of Cx43 in the Mfn-DKO hearts. Cross-sections of left ventricle from 8-weeks-old males, (**A**) WT control versus (**B**) Mfn-DKO, cardiac-specific, transgenic mice, immuno-stained with anti-Cx43 (brown). Examples of lateralised regions of Cx43 are delineated with red arrows. The negative controls using normal rabbit IgG and secondary antibody solely, without primary antibody, are represented by (**C,D**) respectively. Scale bar = 20 µm (**E**) Quantitative analysis of Cx43 lateralization. Images are representatives of 3 hearts for each group. Data are presented as mean ± SEM; *p < 0.05 when compared against WT.

Given that CV in the Mfn-DKO hearts was reduced compared to the WT hearts, we next evaluated the lateralization of Cx43 as this has been linked to gap junction function and cardiac conductivity. IHC staining of ventricular tissue showed that the Cx43 in WT mice was confined to the gap junctions (Fig. 4A), whereas localization of Cx43 in the Mfn-DKO was predominantly lateralized (Fig. 4B), a finding which is consistent with the reduced CV we observed in the Mfn-DKO hearts, when compared to WT ones. Figure 4C,D serve as the negative control with staining using normal rabbit IgG antibody and secondary antibody without the primary antibody, respectively. Figure 4E shows the significant increase in Cx43 lateralization against total Cx43 in the cardiac tissue.

### Lateralization of Cx43 was not associated with phosphorylation at Ser368

**Figure 5:**
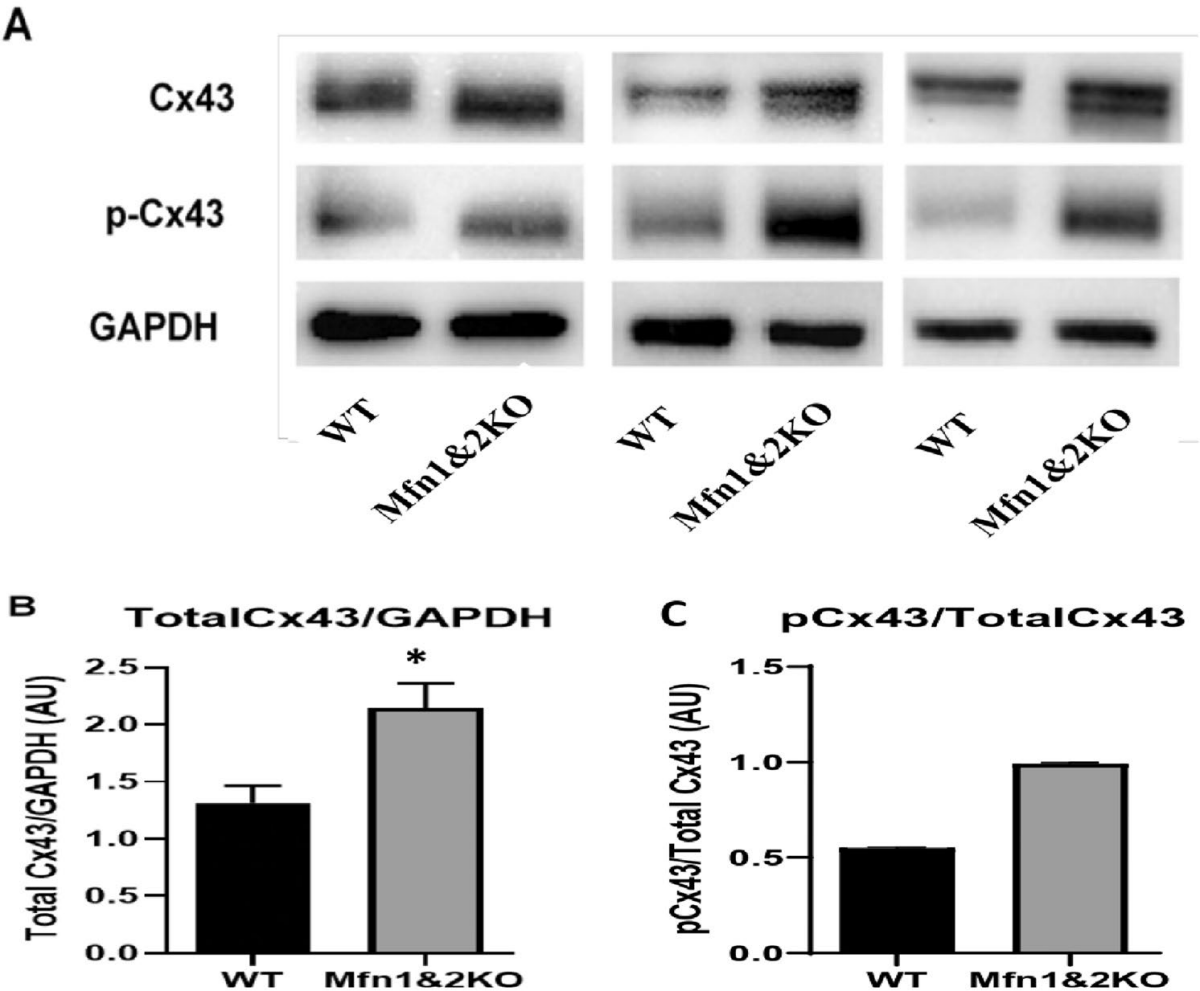
Cx43 protein expression in the Mfn-DKO versus WT hearts. (**A**) Representative immunoblots (cropped) of total Cx43 and phosphorylated Cx43 from 3 different WT versus Mfn-DKO hearts. Original gel images are presented in Supplementary Fig. 2. (**B**) Quantification of band intensity for total Cx43, normalized to GAPDH expression, *p < 0.05 when compared against WT. (**C**) Quantification of band intensity for phosphorylated Cx43, normalized to total Cx43.

The de-phosphorylation of Cx43 at Ser368 has previously been associated with the lateralization of Cx43 in the heart^36,37^. Total Cx43 normalized against GAPDH was significantly increased in the Mfn-DKO hearts compared to the WT hearts (Fig. 5A,B). Densitometry analyses demonstrated no significant difference in the expression levels of phosphorylated Cx43 at Ser368 between the hearts of WT versus Mfn-DKO mice when normalized against the total Cx43 (Fig. 5A,C).

## Discussion

Our observations indicate that the genetic ablation of both mitofusins in the heart leads to increased APD and reduced CV at baseline, particularly in the left ventricle, when compared to WT heart tissue, suggesting that changes in mitochondrial morphology proteins can impact on cardiac conduction and provide an arrhythmogenic substrate. Following simulated ischemia, CV in the atrial and ventricular tissue of the WT hearts reduced significantly—a phenomenon lost in the Mfn-DKO hearts..Following simulated reperfusion, the CV was restored to pre-ischemic levels in the WT hearts but not the left atria of the Mfn-DKO hearts. As the baseline CV was significantly lower in the left ventricle of the Mfn-DKO hearts when compared to WT hearts, CV values between simulated ischemia and reperfusion in the left ventricle of the Mfn-DKO hearts were not significantly different. We also demonstrated increased lateralization of Cx43 in the Mfn-DKO hearts when compared to WT hearts, although the phosphorylation at Ser368 was not different between the Mfn-DKO hearts versus the WT hearts.

Although our previous study demonstrated increased mitochondrial fragmentation and impaired respiration in the Mfn-DKO mice^38^, there has been no study investigating the susceptibility to cardiac arrhythmias in Mfn-DKO mice. The association between mitochondrial dynamics and cardiac arrhythmias also remains elusive. To the best of our knowledge, this is the first study investigating cardiac APD and CV in Mfn-DKO hearts. The fragmented mitochondria and impaired respiration observed in the Mfn-DKO hearts suggest mitochondrial dysfunction^38^, in which case the disrupted intracellular ion homeostasis and membrane excitability caused by reduced ATP production and excessive ROS may be associated with impaired cardiac electrical functioning, as we detected in the baseline difference in both APD and CV between the DKO-hearts versus the WT hearts. Increased ROS may lead to uncoupling of ATP production and damage to the components of electron transport chain (ETC) during IR^9,39,40^, rendering the ion channels/transporters to malfunction in a vicious cycle leading to further increased APD and corresponding CV reduction in the DKO-hearts.

Another plausible rationale for the reduced CV in the DKO-hearts lies in the varying levels of Cx43, in different sources of tissue (different heart chambers) and across different species. Although the total Cx43 in the DKO increases, there may also be a loss-of-function in the Cx43 in the Mfn-DKO in line with the lateralization of Cx43^41–44^ which could potentially explain the reduced CV observed at baseline, albeit a thorough investigation of the function of Cx43 is warranted. Furthermore, it should also be noted that the level of Cx43 expression may not be directly correlated to CV, a notion further confounded by the regional heterogeneity of Cx43 expression as demonstrated in previous studies^45–47^.

The process of ischemia whereby oxygen is removed alongside respiratory substrates, impairs ATP production and the proper functioning of the ion channels. Myocardial stunning and arrhythmias ensue^48^, a finding supported by the reduction in CV observed following ischemia in both atrial and ventricular tissue in our study. Reperfusion restored the CV significantly compared to the lowest CV observed during ischemia. Cardiac ischemia has also been demonstrated to initiate mitochondrial fragmentation—a phenotype which may persists throughout reperfusion^26,27^. Fragmentation of the mitochondria, which can be mediated by either an upregulation of the pro-fission proteins as well as a downregulation of the pro-fusion proteins, is also associated with elevated ROS leading to a net increase in [Ca^2+^]_i_ in cardiomyocytes^45,46^ and induction of fibrosis^49^. Mff-mediated mitochondrial fission during acute cardiac IR is also associated with excessive ROS production, oxidation of cardiolipin and triggering dissociation of hexokinase 2 (HK2)^50^. Increased oxidative stress renders the RyR2 to maintain an ‘open’ state thus increasing the efflux of Ca^2+^ from SR whilst inhibiting SERCA. This is further exacerbated by the reversal of the mitochondrial NCX thus pumping more Ca^2+^ into the cytosol in line with depletion of the SR Ca^2+^ store in a process termed calcium-induced calcium release. This perturbed Ca^2+^ homeostasis in combination with ischemia-mediated changes to intracellular pH and cellular cAMP have also been known to reduce OPA1 expression and prompt calcineurin-mediated Drp1 phosphorylation and mitochondrial fragmentation^51^ while mediating gap junction uncoupling, lateralization of Cx43 and impedance of CV^47,52,53^.

The propensity towards cardiac arrhythmogenesis is further exacerbated during reperfusion when the mitochondrial metabolic substrate intermediates accumulated during ischemia are released as ROS, further damaging the L-type Ca^2+^ channel, Na–K ATPase and SERCA whilst disrupting the electron flow and mitochondrial membrane potential^54^. In addition, the properties of the ion channels as well as the physical state of the cardiomyocytes and their interconnections also determine CV. A reduced CV is associated with an increased probability of re-entry circuits excitation, predisposing to cardiac arrhythmia^55^. Different factors may lead to a reduction in CV such as the changes in action potential duration (APD) mediated by the different ion channels, decoupling of gap junctions and the resulting increase in axial resistance as well as a possible mis-alignment of myocyte-myocyte positioning^56,57^. Studies have reported the PKC-dependent phosphorylation of Cx43 at Ser368 to be associated with decreased gap junctional communication and conductance^58^ while a PKA-dependent de-phosphorylation of Cx43 at Ser368 causes impaired cardiac CV^14,15^, thus indicating that different upstream kinases may exert varying effects on the phosphorylation status of Cx43^59^. Genetic ablation of Cx43 as well as reduced phosphorylation of Cx43 during ischemia leading to lateralization of Cx43 reduces CV^60,61^. All of these predisposes to arrhythmogenesis by increasing the APD, delayed repolarizations and ensuing contractile dysfunction. However, our results indicate that the level of phosphorylated Cx43 at Ser368 normalized to the total Cx43 in the Mfn-DKO hearts is not different compared to the WT hearts. A potential explanation is the assembly, trafficking and turnover of Cx43 is governed by phosphorylation at different carboxyl-terminal serine and threonine residues, which in turn renders varying downstream intercellular electrical and metabolic coupling effects in the heart^62–67^. Although not investigated in our study, the prevalence of a particular PKA or PKC isoform during ischemia–reperfusion in the Mfn-DKO hearts may also dictate the phosphorylation status of Cx43 at Ser368. The lateralization of Cx43 may also be due to the Cx43 being in the process of being trafficked to the intercalated disks to compensate for impaired gap junctions and lost cellular connectivity^68^. Another possibility is the activation of Src kinase which has been known to occur under certain conditions of stress such as during hypoxia or ischemia. Src will bind to the scaffolding protein ZO-1 at the ID thereby causing Cx43 to lateralize as it is unable to bind to the scaffolding protein at the gap junction^69,70^. However, our study did not investigate the levels of the Src kinase. Perturbations in the interaction of Cx43 with the actin cytoskeleton has also been demonstrated to lead to mis-localization of Cx43 although this was not investigated in our study^71^.

In summary, we have shown that the left ventricle of the Mfn-DKO hearts have reduced CV compared to the WT hearts at baseline level. This difference in CV between the Mfn-DKO hearts and WT hearts persisted throughout ischemia and reperfusion, albeit more significant in the left ventricle. Of interest, reperfusion reverses the ischemia-induced decline in CV in the ventricle tissue but not the left atrial tissue of the Mfn-DKO hearts. Our results propose a link between the mitofusins, mitochondrial dysfunction, and arrhythmia susceptibility while highlighting the potential therapeutic effect of modulators of mitochondrial morphology in mitigating cardiac arrhythmias. The limitations of this study include a low number of animals studied, a lack of sex-specific phenotypes with regards to susceptibility to arrhythmias, the absence of in vivo arrhythmic investigation in the Mfn-DKO mice, lack of direct evidence between reduced CV and lateralization of Cx43 as well as corroboration between phosphorylation status at different sites of Cx43 in mediating lateralization of Cx43. Ideally, patch clamp experiments should also be conducted in the isolated cardiomyocytes to determine whether a reduced expression of cardiac sodium channels (Nav1.5) or a partial inactivation of Nav1.5 channels due to the metabolic alteration in the Mfn-DKO mice leading to a less negative resting membrane potential may contribute to conduction slowing. A better standardization of the number of hearts/cells used across WT versus Mfn-DKO mice and heart chambers will also increase the robustness of the data.

The ventricular preparation procedure has some limitations. The transverse mid-ventricular section comprises of largely circumferential orientated myofibers. Only short-axis ventricular sections were analyzed and myofiber orientation was not delineated. This has implications on anisotropy which is a recognized feature of cardiac conduction properties with faster conduction velocity along the long axis as compared to the short axis which is not assessed. Linear propagation of conduction was assumed which may be an oversimplification of the conduction path. Longitudinal or epicardial conduction properties were not assessed due to equipment limitations to maintain electrode contract on the immobile MEA platform.

Future work includes assessing the expression level, phosphorylation status and localization of Cx43 in the cardiac tissue post-ischemia reperfusion. Further in vivo studies are also warranted to investigate the susceptibility to cardiac arrhythmias in the Mfn-DKO mice at baseline as well as post-ischemia–reperfusion.

## Materials and methods

### Animal model

All animal procedures were approved by the SingHealth Institutional Animal Care and Use Committee (2015/SHS/1049) conforming to the National Advisory Committee for Laboratory Animal Research (NACLAR) guidelines for the Care and Use of Animals for Scientific Purposes. All mice were maintained in individually ventilated cages, with free access to standard rodent chow and water under 12-h day-night cycles. To generate conditional cardiomyocyte-specific ablation of Mfn1 and Mfn2, Mfn1^loxp/loxp^ and Mfn2^loxp/loxp^ double knock-out (Mfn-DKO) mice courtesy of Gerald W. Dorn II (Washington, St Louis, MO USA) were crossed to Myh6-MerCreMer inducible mice as previously described^38,72^. Cardiomyocyte-specific ablation was initiated in 4–6 week-old male Mfn-DKO mice with 5 consecutive days of tamoxifen administration (IP.; 20 mg/kg/day). Control mice (WT) were flx/flx littermates injected with tamoxifen but lacking the Cre-recombinase gene. Mice aged 8–10 weeks were euthanized at endpoint with ketamine (100 mg/kg) and xylazine (10 mg/kg) given IP and heart removed under deep anesthesia.

### Buffer solutions

The following Tyrode’s master solution was made up prior to slice preparation on the day of procedure: NaCl (155 mM), KCl (5.4 mM), NaH_2_PO_4_ (0.33 mM), glucose (10 mM), HEPES (10 mM), 2,3-butanedione monoxime (BDM) (30 mM), MgCl_2_ (1 mM) dissolved in 1 L of distilled water. This ‘Ca^2+^-free Tyrode’s’ solution was then used to produce the following modified Tyrode’s solution by varying the CaCl_2_ concentration: (1) Incubation Tyrode’s (Ca^2+^ 0.9 mM) at 21 °C, (2) Equilibrium Tyrode’s (Ca^2+^ 1.8 mM) at 37 °C, and (3) Hypoxic Tyrode’s (Ca^2+^ 1.8 mM) gassed with nitrogen gas (N_2_) for 30 min at 37 °C. Incubation and Equilibrium Tyrode’s solution was bubbled with pure oxygen.

### Optical electrophysiology for measurement of action potential duration (APD)

Optical-electrophysiology was performed as outlined previously^73^ using a voltage sensitive FluoVolt dye (Thermo Fisher Scientific, USA), and the absolute fluorescence signals were obtained with a custom built novel high-throughput imaging platform (OptioQUANT) (Ternion Bioscience, Singapore). All data were analyzed with Igor Pro (WaveMetrics, USA). AP durations of WT and Mfn 1&2 KO mouse cardiomyocytes were measured at 30%, 50%, 70% and 90% decrement of AP amplitude and analyzed. Cells which exhibited irregular events during acquisition were excluded from analyses. All values are given as mean ± SEM.

### Multi-electrode array (MEA) for measurement of cardiac conduction velocity (CV)

CV of ex vivo murine left atrial and ventricular tissue preparations was conducted using a MEA as previously described^74–76^. Mouse hearts were removed from the anesthetized Mfn-DKO or WT mice and rinsed with ice-chilled Ca^2+^-free Tyrode’s solution. The left atria was carefully dissected for direct emplacement with the epicardial surface onto the electrodes of the MEA chip for electrophysiology studies. The ventricular apex was transected transversely and molded in 4% agarose. The agarose-embedded ventricle was mounted on the vibratome (Leica, VT1000S) for sectioning tissue slices at 250 µm. The slices were then transferred to Incubation Tyrode’s solution for 30 min, and subsequently to Equilibrium Tyrode’s solution for another 30 min until electrophysiology studies were performed.

**Figure 6:**
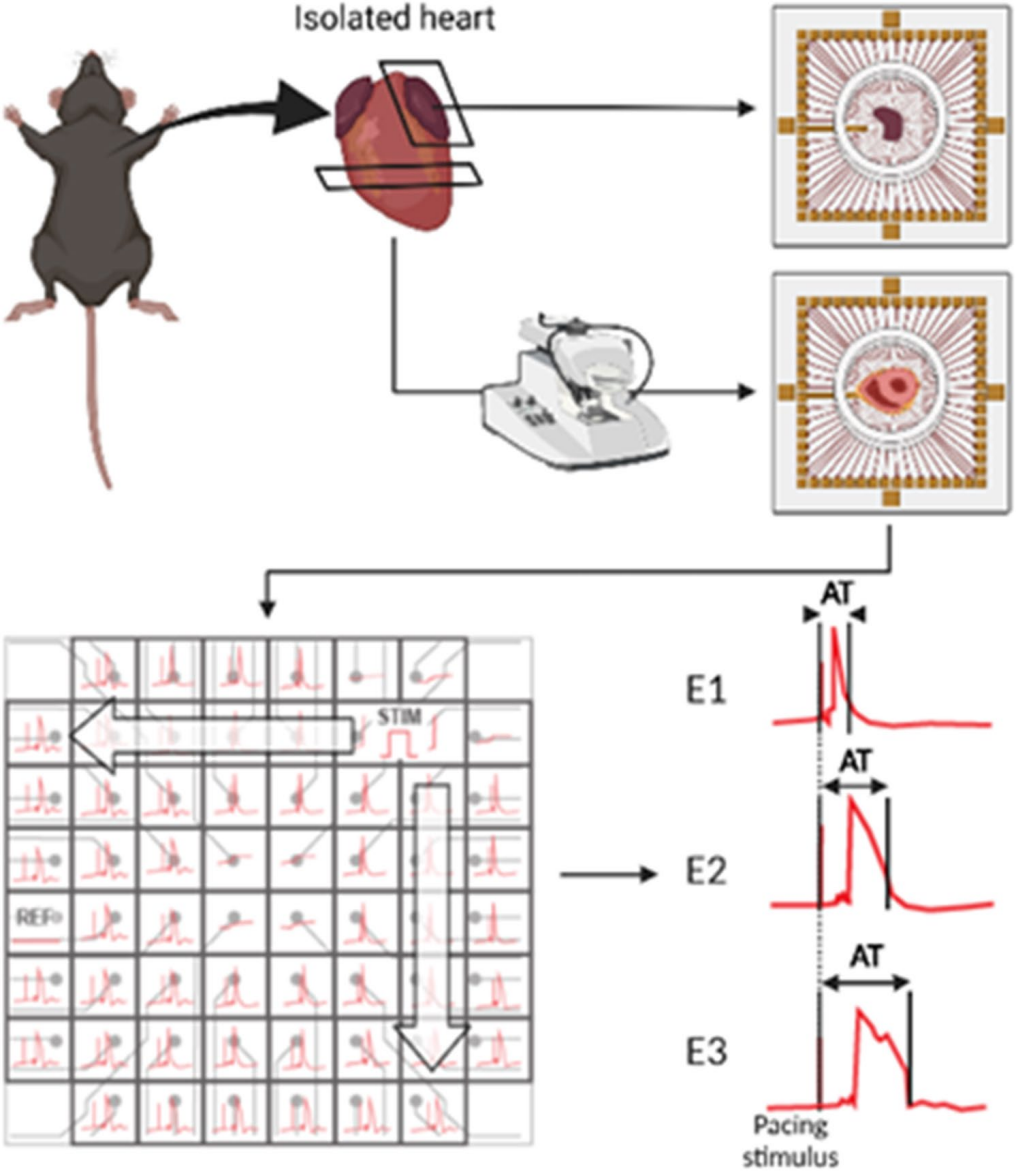
Schematic of murine atrial and ventricular isolation for MEA studies. Murine atrial tissue were placed epicardial surface down whereas transverse cross-section of the ventricle was emplaced onto the MEA for electrophysiological studies. Tissues were weighed down by a weighing harp. Representative electrograms recorded from a ventricular tissue slice using the MEA, with the proximal electrode (E1) and progressively distal electrodes (E2 and E3) to the paced stimulus. STIM indicates site of pacing stimulus, REF indicates the reference electrode position of the MEA. AT indicates activation time measured from stimulus onset to the steepest negative deflection of the electrogram. Created with BioRender.com”.

Left atrial and ventricular CV were assessed during electrical stimulation using the MEA system allowing for non-invasive synchronous multifocal recording of unipolar electrograms (UEGMs) as previously described (Fig. 6)^74,75^. The MEA chip (MultiChannel Systems, Pedot MEA) consists of 59 electrodes and one reference electrode, arranged in an 8 × 8 matrix (Atrial: 20 µm electrode diameter and 200 µm interelectrode distance; Ventricular: 100 µm electrode diameter and 700 µm interelectrode distance). The chip was loaded onto the amplifier connected to the powerpack (MultiChannel System, PS40W) and the whole MEA chamber was continuously perfused by a peristaltic pump (Harvard apparatus, MPII) with oxygenated Equilibrium Tyrode’s solution (2 mL/min). Atrial tissue and ventricular slices were emplaced onto the MEA chip and weighed down with a 1-cm diameter nylon mesh harp slice grid to ensure adequate contact with the electrodes. For consistency purposes, the left atrial and ventricular tissue was maintained in the same orientation throughout. Additionally, the edges of the left atria/ventricle was positioned to maintain contact with the same stimulation electrodes (62 and 72) in all experiments.

After 15 min of stabilization on the MEA chip, electrical stimulation (biphasic pulses, twice current threshold, 2 ms duration, 4 Hz frequency) was applied to the heart slices at the same stimulation electrodes using MC Stimulus (MultiChannel System). UEGMs were acquired simultaneously from all 60 microelectrodes and feedback responses captured by MC_Rack (MultiChannel System). The baseline reading was recorded in the presence of oxygenated Equilibrium Tyrode’s solution at 37 °C. To simulate IR conditions ex vivo, we varied the physiological buffer with oxygen or nitrogen supplementation. Atrial tissue or cardiac slice was challenged with hypoxic conditions by replacing the oxygenated Equilibrium Tyrode’s solution with oxygen-deprived Hypoxic Tyrode’s solution for 8 min and then re-exposed to oxygenated Equilibrium Tyrode’s solution for 32 min^77^. To access conduction properties, the tissue samples were stimulated at 4 Hz and readings were obtained at 2-min intervals starting from 0 to 40 min. Analysis was performed offline using Clampfit (Molecular device). The time difference between pacing stimulus and steepest negative deflection on the UEGM was defined as the local activation time and average CV was calculated using interelectrode distance and the difference in activation times^74,75^.

### Western blotting

Cardiac tissue samples were prepared in lysis buffer containing 50 mm Tris–HCl, 150 mm NaCl, 1 mm EGTA, 1 mm EDTA, 1% Triton X-100, and protease inhibitor mixture (Roche Applied Science) and run on Invitrogen NuPAGE Bis–Tris gels. The protein expression levels in the cardiac tissue were analyzed using standard western blotting techniques with the following antibodies for Mfn1 (Ab57602, Abcam, 1:1000 fold dilution), Mfn2 (9482S, Cell Signalling, 1:1000 fold dilution), Cx43 (3512S, Cell Signalling, 1:6000 fold dilution), phospho-Cx43 (48-3000, Invitrogen, 1:1000 fold dilution), GAPDH (60004-1-1g, Proteintech, 1:5000 fold dilution).

### Histology and immunohistochemistry

Histology and immunohistochemistry were performed as described previously^78^. Briefly, heart tissue was harvested and fixed overnight in 4% paraformaldehyde at 4 °C. Subsequently, the samples were dehydrated, processed for paraffin embedding and sectioned at 4 μm. Microscopic slides underwent heat antigen-retrieval, blocked with 5% BSA and incubated with anti-Cx43 (1:100; CST, 3512S) overnight at 4 °C. Sections were then washed with tris-buffered saline with Tween 20 and incubated with a secondary antibody (1:1000; CST, 7074S) at room temperature for 2 h. HRP activity was detected either by using a DAB kit (Vector Laboratories, SK-4100). Sections were mounted with DPX mounting medium for observation under the light microscope. At least five different regions of each section were studied by two blinded operators. Lateralization of Cx43 was scored as a percentage against total Cx43 in the images.

### Statistics

All data are presented as mean ± SEM. Results were analyzed using an unpaired t-test for comparison between two groups. For conduction velocity assessment across time in atrial and ventricular tissue following simulated ischemia–reperfusion in WT and Mfn-DKO mice, a two-way repeated measures ANOVA with Sidak multiple comparisons was performed. Statistical significance was achieved when P < 0.05.

### Institutional review board statement

The study was conducted according to the guidelines of the Declaration of Helsinki, the ARRIVE guidelines and approved by SingHealth Institutional Animal Care and Use Committee (2015/SHS/1049—approval date 2015).

## Supplementary Information

Supplementary Figures.

## Data Availability

The data presented in this study are available on request from the corresponding authors.
