# Supplementary material for: Role of cardiac mitofusins in cardiac conduction following simulated ischemia–reperfusion
Source: Sci Rep. 2022 Dec 6;12:21049. doi: 10.1038/s41598-022-25625-0 (PMC9727036; doi:10.1038/s41598-022-25625-0)

SUPPLEMENTARY FIGURE 1

Original blots for Figure 1A

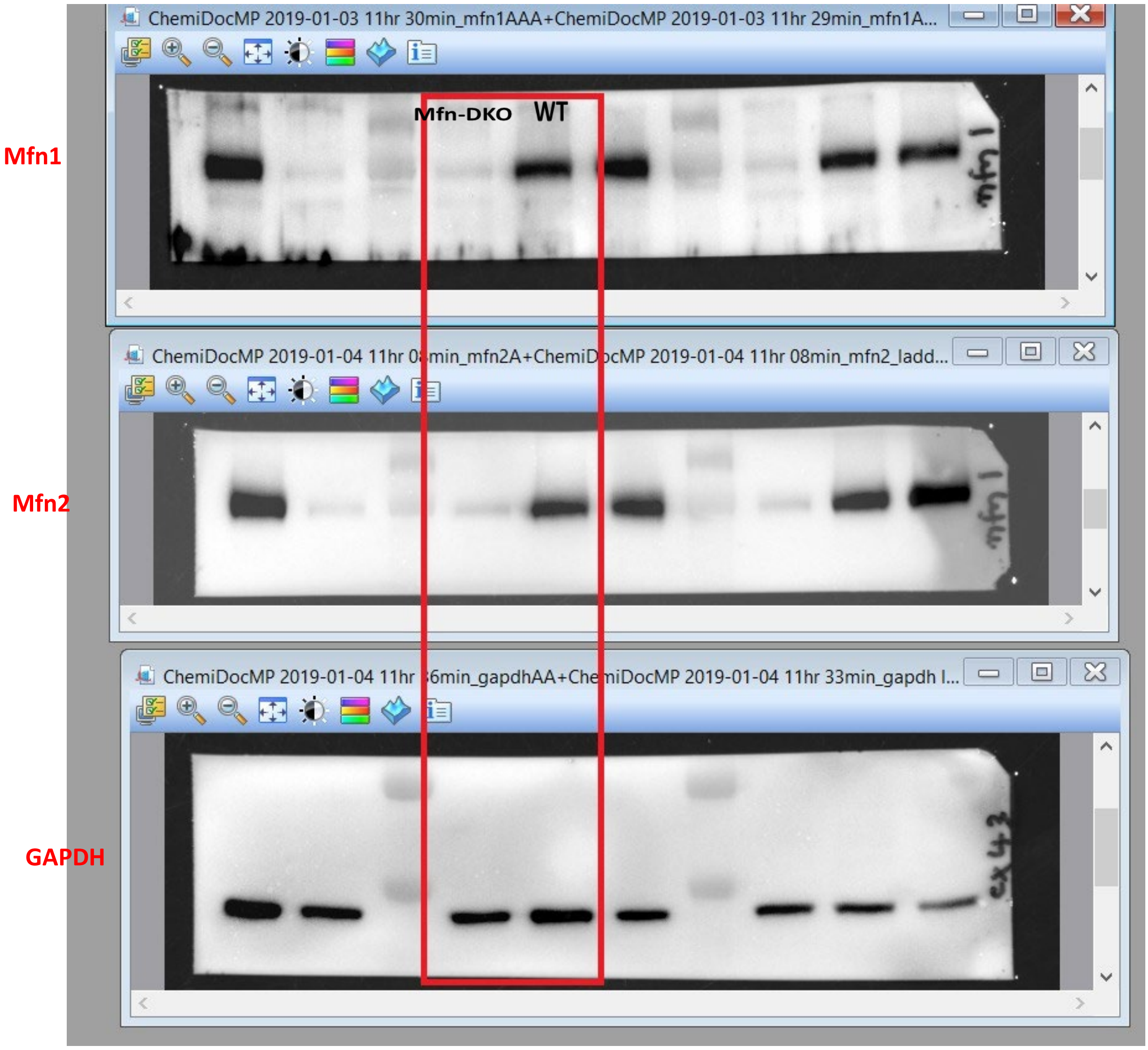

SUPPLEMENTARY FIGURE 2

Original blots for Figure 5A

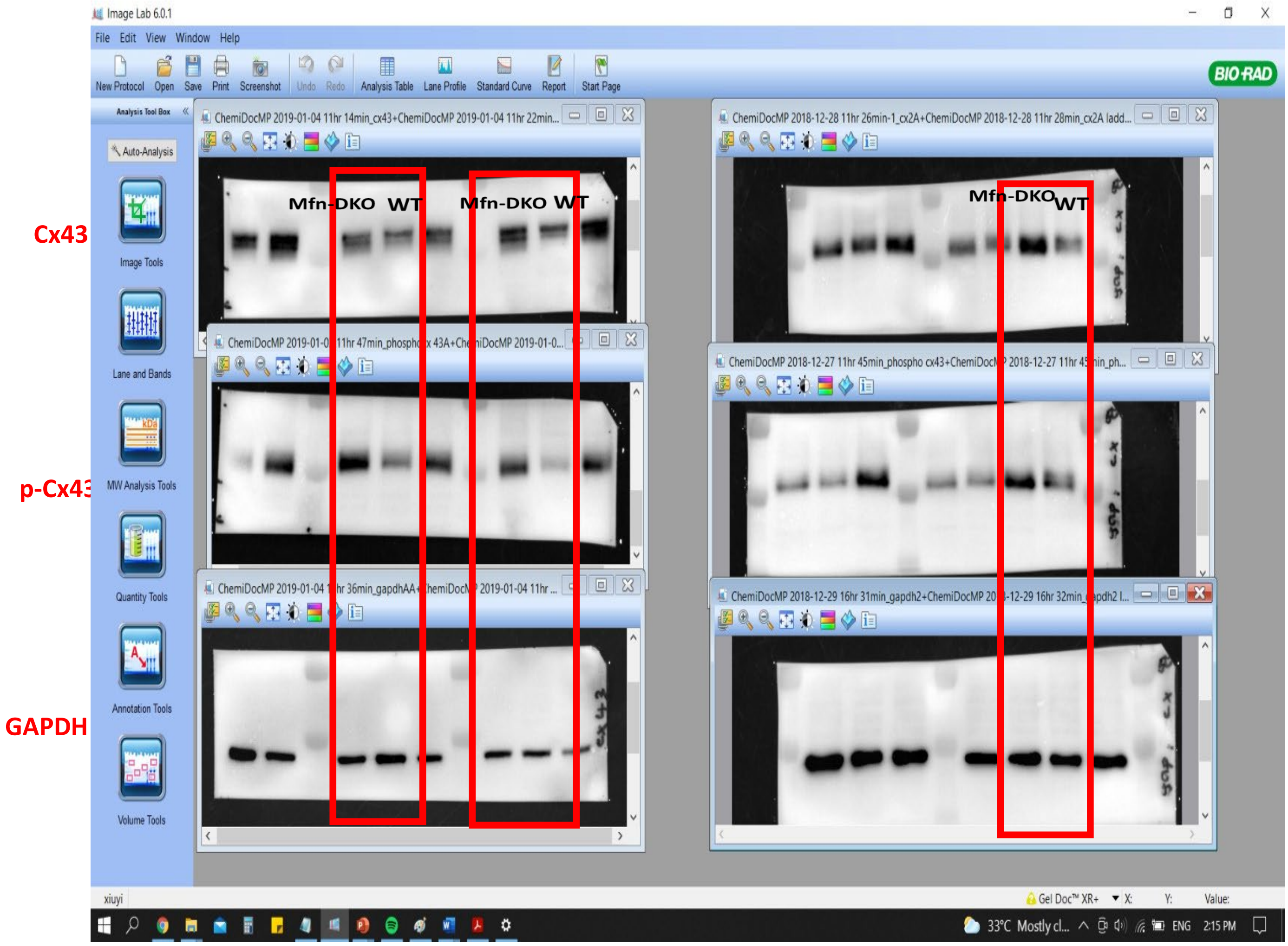

Supplement: Supplementary file 1 — Supplementary Figures. [file 41598_2022_25625_MOESM1_ESM.pdf]
